# Effect of Nonconstituent Additive Ions on the Controlled Crystallization of Lanthanide-Based Preyssler Polyoxometalates

**DOI:** 10.1021/acs.cgd.3c00046

**Published:** 2023-04-06

**Authors:** Iván Gómez-Muñoz, Chandan Dey, Eugenio Coronado

**Affiliations:** Instituto de Ciencia Molecular (ICMol), Universidad de Valencia, c/Catedrático José Beltrán 2, Paterna 46980, Spain

## Abstract

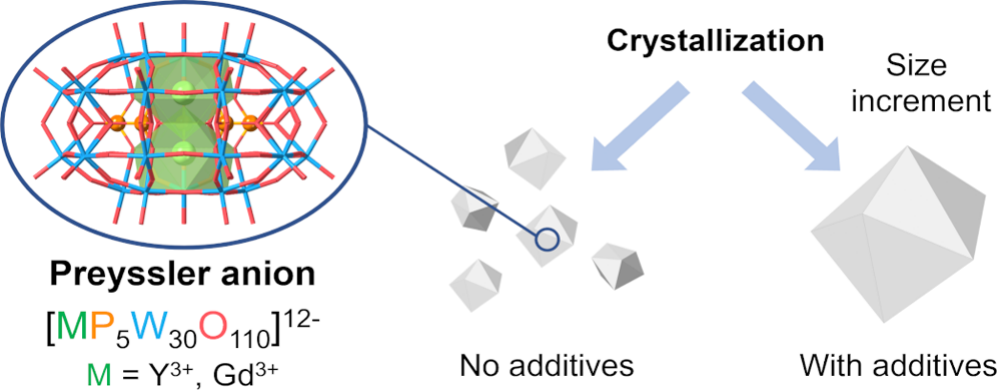

Preyssler-type polyoxometalates (POMs) encapsulating lanthanide ions have been shown to provide ideal examples of single-molecule magnets and spin-qubits. However, the advances in this area are limited by the quality and size of the crystals. In this work, the role of additives ions in the crystallization of these POMs from aqueous solutions has been investigated. More specifically, we have studied the influence of Al^3+^, Y^3+^, and In^3+^ on the crystallization process of K_12_[MP_5_W_30_O_110_] (where M = Gd and Y). The results show that the concentration of these ions in the solution plays an important role in controlling the crystallization rate of the grown POM crystals leading to a significant increase in their size, while showing very little or no tendency to be incorporated into the structure. This has allowed us to obtain pure Gd or Y crystals, as well as diluted magnetic crystals formed by the diamagnetic Y^3+^ POM doped with the magnetic Gd^3+^ ion.

## Abstract 2

The crystallization of the Preyssler anion in the presence of additional cations leads to the formation of bigger crystals in comparison to the crystallization performed with no additives. The crystallization rate and the size depend on the concentration of the additive in the solution during the crystallization.

## Introduction

Polyoxometalates (POMs) comprise a wide range of metal-oxide cluster materials which impact diverse areas of current interest such as catalysis, medicine, materials science,^1−6^ and magnetism,^7,8^ to name a few. Amidst all the different types of POMs, the Preyssler-type anion was discovered in 1970^9^ and presents the general formula [M^*n*+^P_5_W_30_O_110_]^(15–*n*)–^ (shortened as MW_30_, where M = Na, Y, Gd, Tb, Dy, etc.). This structure consists of an anionic cluster, [P_5_W_30_O_110_]^15–^, which can encapsulate one metal ion in the framework POM structure.^10,11^ The charge-compensating counterions consist typically of NH_4_^+^, K^+^, or tetrabutylammonium (TBA), based on the desired solubility. When the encapsulated metal is a lanthanide, this type of POM has been shown to be of great interest in molecular magnetism and quantum computing since it can afford unique examples of single-molecule magnets^12,13^ and spin qubits.^14,15^ However, these current applications are limited by the stability and size of the crystals, as often good quality crystals are required to perform detailed physical measurements.

Studies related to the effect of additives on the crystallization of polymorphs of POMs are only a few. The crystallization of [ε-Al_13_O_4_(OH)_24_(H_2_O)_12_]^7+^ and Keggin-type anion yields a polymorphic ionic salt when the process is carried out in the presence and absence of NaCl.^16^ More recently, the influence of nonbridging cations during the formation of coordination polymers with a Preyssler-type POM and Co^2+^ has been studied.^17^ To the best of our knowledge, the effect of additive ions on the controlled crystallization of POMs has not been explored to date. Thus, in the present work, we have investigated the role of additional nonconstituent ions (Al^3+^, Y^3+^, and In^3+^) on the crystallization of Preyssler-type POMs focusing in particular on GdW_30_ and YW_30_ derivatives. These salts have been chosen as representatives of trivalent nonmagnetic species since even if they are incorporated into the structure, they will be innocent from the magnetic point of view. This study will explore the influence that these ions have on the crystallization rate, the crystal size, and the crystal structure.

## Experimental Section

All reactants and solvents are commercially available and were used as received without any additional purification.

### Synthesis and Crystallization

Preyssler-type POMs, K_12_[MP_5_W_30_O_110_]·*n*H_2_O (M^3+^ = Y, Gd), were prepared according to a previously described method^11^ consisting of a solvothermal reaction of the preformed K_14_[NaP_5_W_30_O_110_]·*n*H_2_O with the selected M^3+^ salt in water. The recrystallization in the presence of metal ions was carried out by the following procedure: 0.5 mL of a 0.6 M metal salt solution (AlCl_3_, YCl_3_·6H_2_O or InCl_3_·4H_2_O) was added to 1 mL of a 0.03 M POM solution. The mixture was heated at 80 °C for 15 min and filtered, letting it sit at ambient conditions in a 10 mL vial covered with parafilm with small holes. After several days, the formation of crystals was observed. For a blank experiment without the presence of any additional salt, 0.5 mL of milli-Q water was added to 1 mL of a 0.03 M solution of POM.

### Characterization

The obtained compounds were characterized by inductively coupled plasma mass spectrometry (ICP-MS), thermogravimetric analysis (TGA), and powder and single-crystal X-ray diffraction (PXRD and SCXRD, respectively). TGA was performed using a TGA 550 (TA Instruments) at a heating rate of 0.3 °C/min from 25 to 120 °C under a nitrogen atmosphere. PXRD experiments were acquired on an X-ray diffractometer (PANalytical Empyrean) with copper as a radiation source (Cu Kα, 1.5418 Å). For SCXRD measurements, suitable crystals were selected from the aqueous solutions and measured under nitrogen flow (120 K) in an Oxford Diffraction diffractometer equipped with a graphite-monochromated enhanced (Mo) X-ray source (λ = 0.71073 Å). Data collection, unit cell refinements, and data processing were carried out using the CrysAlisPro software package developed by Agilent Technologies. The structure solution and refinement were carried out in Olex2 using ShelXT and ShelXL-2018 program, respectively.^18−20^

## Results and Discussion

### Crystallization in the Presence of Additive Ions

In general, it is observed that in the presence of the selected salts (AlCl_3_, YCl_3_, InCl_3_), the rate of crystallization becomes slower and the size of the crystals obtained is bigger compared to the blank experiment. This can be attributed to the loss in the surface energy of the crystal due to the presence of the additives, as well as a partial inhibition of the nucleation by their interaction with the solute.^21,22^ During the crystallization in the presence of AlCl_3_, some white powder is formed on the surface of the crystals which is not possible to isolate, while no additional precipitation was perceived when using YCl_3_ and InCl_3_ additives. The possible incorporation of the added metal ions in the crystal lattice was studied by employing ICP-MS. The results of the analyses (Table 1) show that the amount of insertion of the additive ions is negligible, except in the case of Y^3+^, where the incorporation is comparatively higher.

**Table 1:** Elemental Analyses of Each Compound Performed with ICP-MS

starting POM	additive	composition
GdW_30_	In^3+^	K_11.8_In_0.06_[GdP_5_W_30_O_110_]·35H_2_O
	Y^3+^	K_11_Y_0.33_[GdP_5_W_30_O_110_]·35H_2_O
YW_30_	In^3+^	H_2_K_10_In_0.03_[Y_0.7_Na_0.3_P_5_W_30_O_110_]·60H_2_O
	Al^3+^	K_12.5_Al_0.09_[Y_0.7_Na_0.3_P_5_W_30_O_110_]·30H_2_O

### Structural Analysis and Crystal Phases

**Figure 1:**
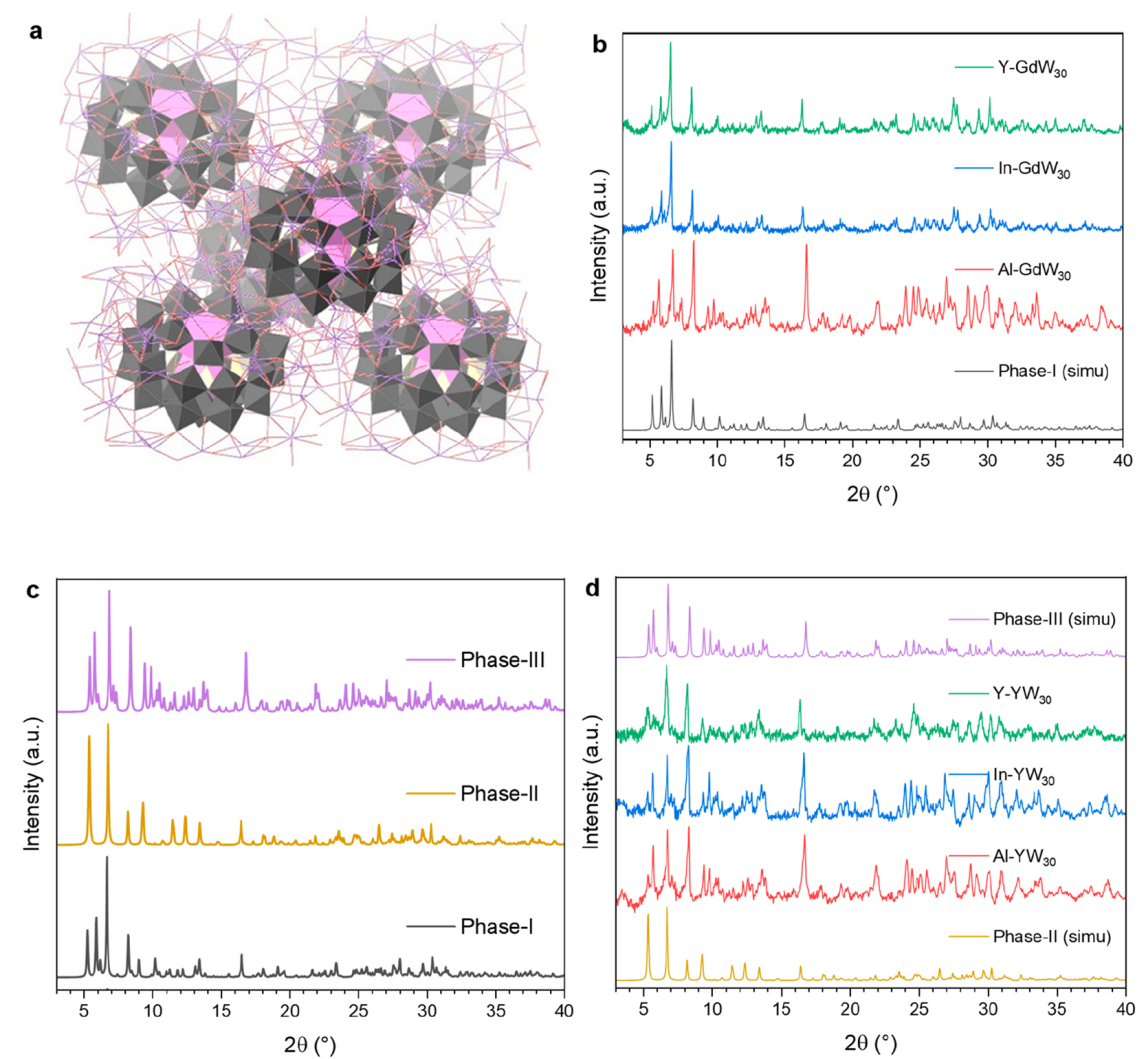
(a) Crystal structure of K_12_[GdP_5_W_30_O_110_]·*n*H_2_O. (b) Experimental PXRD patterns of GdW_30_ grown in the presence of Al^3+^ (red), In^3+^ (blue), and Y^3+^ (green) and comparison with the simulated pattern of Phase-I (black). (c) Comparison of the simulated PXRD patterns of the different crystal phases obtained: Phase-I (black), Phase-II (yellow), and Phase-III (purple). (d) Experimental PXRD patterns of YW_30_ grown in the presence of Al^3+^ (red), In^3+^ (blue), and Y^3+^ (green), and comparison with the simulated diffractograms of Phase-II (yellow), and Phase-III (purple).

In a regular method, pure K_12_[GdP_5_W_30_O_110_]·*n*H_2_O crystallizes in the *Pnma* space group of the orthorhombic crystal system [*a* = 28.5872(4), *b* = 21.4797(3), *c* = 20.8888(3) Å; α = β = γ = 90°], hereafter called as Phase-I (**P-I**) (see Table S1). The symmetric unit contains one-half of the Preyssler-type POM cluster, six K^+^ ions, and 11.75 water molecules (although the total number of water molecules is batch-dependent). Within the Preyssler cluster, the Gd^3+^ ion is disordered in two equivalent positions in the cluster. A typical Preyssler-type POM contains five W atoms in both outer rings, giving rise to a *C*_5_ symmetry axis. The POM units are located in the frame of scattered K^+^ ions, which mostly have partial occupancy in each position (Figure 1a). The small amount of the additive metal ions detected by ICP-MS experiments is undetectable by X-ray crystallography. The crystalline phase of GdW_30_ obtained in the presence of In^3+^ and Y^3+^ is the same as that of the original phase (**P-I**). This is confirmed by checking the unit cell parameters of selected single crystals and comparing the simulated X-ray diffraction pattern of the samples with the experimental ones (Figure 1b). However, a phase change is introduced in the single crystal form when the same experiment is carried out in the presence of Al^3+^. In this case, the unit cell parameters are *a* = 32.8072(3), *b* = 21.5301(2), *c* = 19.1367(2) Å; α = β = γ = 90°, and the space group is *Pnna* (see Table S1). This crystal phase will be referred to as Phase-II (**P-II**) hereafter. Nevertheless, the simulated powder pattern of Phase-II does not match the one obtained in the experimental measurements of Al-GdW_30_ (Figure 1c), looking instead more similar to the crystal phase obtained in the crystallization of NaW_30_ in the presence of Al^3+^. The parameters of this third phase, which will be referred to as Phase-III (**P-III**) from now on, are *a* = 16.8636(2), *b* = 21.0688(2), *c* = 18.0295(2) Å; α = γ = 90°, β = 114.859(2)°, and the space group is *P*2_1_/*m* (see Table S1). The observation of the **P-III** diffractogram in the experimental PXRD of Al-GdW_30_ suggests that there could be two different types of crystals growing during the crystallization process (**P-II** and **P-III**). The **P-II** powder pattern could very easily appear hidden in the **P-III** pattern in the experimental measurements, due to the many coincident peaks that both phases present and to the rather noisy diffractograms. On the other hand, the obtainment of **P-II** in all the SCXRD measurements could be explained by the higher quality of these crystals in comparison with the **P-III** ones, which would make them better candidates to be chosen for the experiments. This very same case is observed in the recrystallization of YW_30_ in the presence of the additive ions, as well. In all cases, **P-II** is obtained in SCXRD while the pattern of **P-III** is the one observed in PXRD (Figure 1d).

### Effect of the Concentration of In^3+^

**Figure 2:**
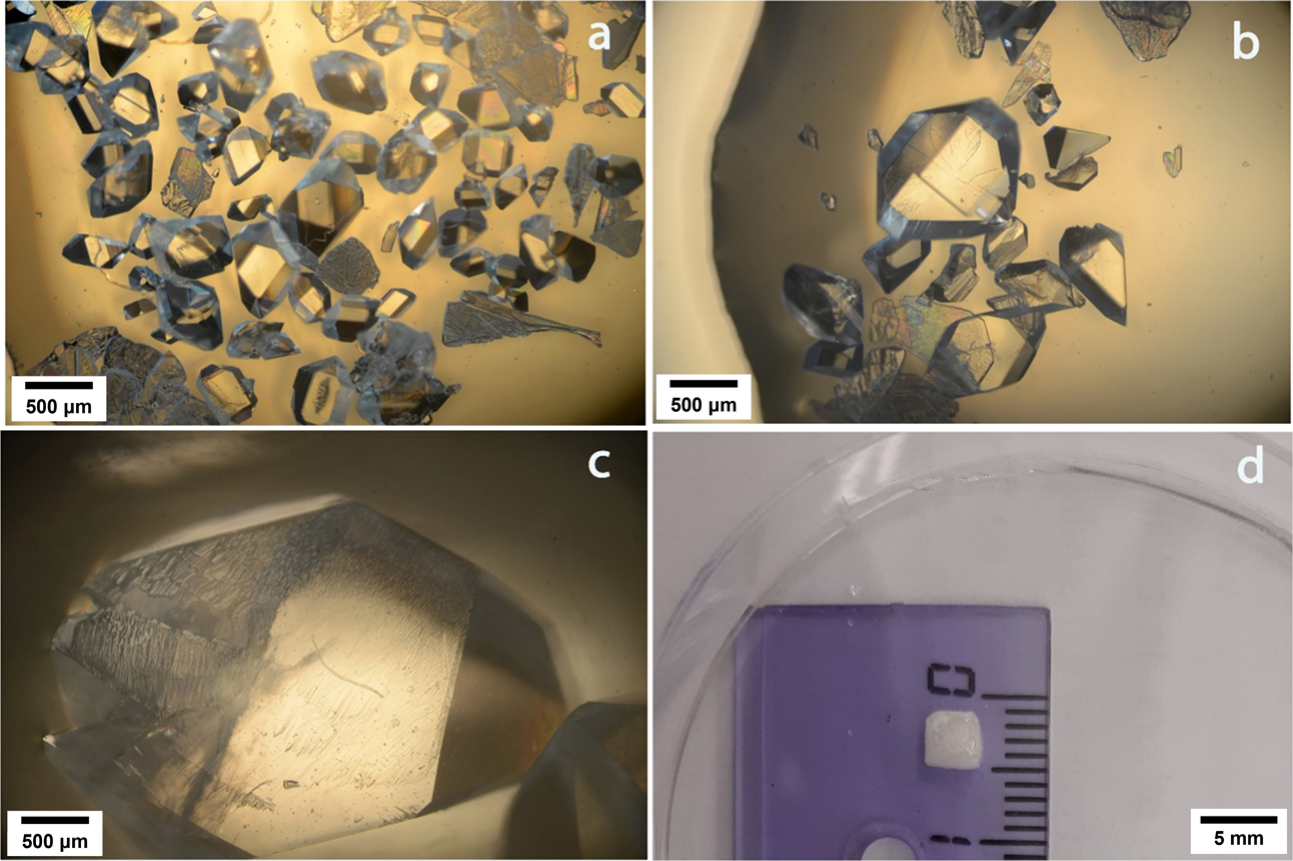
Images of YW_30_ crystals grown in the presence of (a) no In^3**+**^, (b) 0.06 M In^3+^, (c) 0.12 M In^3+^, and (d) 0.18 M In^3+^.

As both Al^3+^ and Y^3+^ additives lead to unwanted results (the presence of additional precipitate in the crystallization of Al-MW_30_ systems and the easy incorporation of Y^3+^ in the structure), we have focused on In^3+^ for further studies since it affords the best results. The effect of the concentration of InCl_3_ on the crystallization of Preyssler-type POM YW_30_ has been investigated in a wide range of concentrations of the salt (from 0 to 0.30 M) (Table 2). It has been observed that when the concentration of InCl_3_ increases, the time of formation of the first crystal is longer and, whereas the total number of crystals obtained is reduced and their size increases along with the concentration (Figure 2). Hence, we can conclude that the presence of InCl_3_ hinders the nucleation process of the POM, this effect being accentuated when the concentration increases_._ However, at concentrations higher than ∼0.18 M, the size of the grown crystals is no longer increased. This can be attributed to a kink-blocking effect.^23−25^ In these conditions a slight change in the phase can also be observed in the PXRD pattern (Figure S2). At a very high concentration of InCl_3_ (∼0.3 M), the high saturation leads to the formation of a dense solution by slow evaporation of the solvent. This hinders the crystal growth, and only the formation of very tiny crystals is favored. Additionally, a study on the influence of the exposed surface area on the crystal size was performed and can be found in section 2 of the Supporting Information (Table S2). Interestingly, for all the studied concentrations of In^3+^, the incorporation of the cation in the structure remains negligible, as confirmed by ICP-MS (Table 2). Thermogravimetric analyses (TGA) were performed on the samples (Figure S3), and the measurements showed diverse results due to the different water content of each sample, which is batch-dependent (also seen by ICP-MS). In addition, to generalize the effect of the crystallization in the presence of In^3+^ salts, different LnW_30_ Preyssler-type POMs (Ln = Tb, Dy, Ho, Er) have been grown. These experiments have shown that in the presence of In^3+^, larger crystals were obtained as a consequence of a slower crystallization rate, in the same way as for GdW_30_ and YW_30_ (see section 3 in the Supporting Information).

**Table 2:** Study of the Crystallization of YW_30_ in the Presence of Different Concentrations of InCl_3_

[InCl_3_] (M)	formation of the first crystals (days)	average crystal dimensions (mm^2^)	composition
0.00	1	0.6 × 0.5	
0.06	1–3	1.5 × 1.0	In_0.02_H_2_K_10_[Y_0.7_Na_0.3_P_5_W_30_O_110_]·20H_2_O
0.12	3–5	3.0 × 2.5	In_0.04_H_2_K_10_[Y_0.5_Na_0.5_P_5_W_30_O_110_]·23H_2_O
0.18	5–7	4.0 × 4.0	In_0.03_H_2_K_10_[Y_0.7_Na_0.3_P_5_W_30_O_110_]·60H_2_O
0.24	7–9	4.0 × 4.0	In_0.05_H_2_K_10_[Y_0.6_Na_0.4_P_5_W_30_O_110_]·60H_2_O
0.30	9–11		

### Co-crystallization of YW_30_ and GdW_30_

The retention of the crystal phase when a nonmagnetic POM is doped with magnetic ions is important for the study and comparison of the magnetic properties. In this way, doping a diamagnetic POM system (YW_30_) with a paramagnetic ion (Gd^3+^) is a common strategy used to study the behavior of these POMs as single-ion magnets or as qubits since this magnetic dilution avoids the magnetic interactions between POM anions and improves the spin-relaxation properties.^13,15^ In the case studied here, we have seen that the single crystal phases of YW_30_ and GdW_30_ grown in the presence of In^3+^ are different. Therefore, we wanted to study the ability of the compounds to retain their original crystal phase when the two systems cocrystallized together. Hence, a series of crystallizations of YW_30_ with a variable doping percentage of GdW_30_ using the same concentration of InCl_3_ (0.2 M) was performed. The hybrid system crystallized faster than pure YW_30_, and the unit cell parameters of the crystals were checked by SCXRD, extracting the corresponding phase index percentage. We could observe that **P-II** can be retained with up to 40% GdW_30_ (see Table S4 and Figure S6). Moreover, the cocrystallizations were also performed under different concentrations of InCl_3_, observing that in this case the crystals can be formed at higher concentrations of In^3+^ (up to 0.4 M compared to 0.24 M in the pure systems; see Table S5).

## Conclusion

The study of the presence of additive ions (Al^3+^, Y^3+^, and In^3+^) in the crystallization of Preyssler-type POMs (GdW_30_ and YW_30_) has shown that the crystallization rate is reduced in the presence of these ions. As a consequence, the crystals obtained are bigger. Among the three different ions studied, In^3+^ has resulted to be the best candidate since the other two present some problems: the tendency of the compounds crystallized in the presence of Al^3+^ to precipitate, and the higher tendency of Y^3+^ to be incorporated in the structure. In addition, it has been proven that it is possible to control the size of the crystals by varying the concentration of In^3+^ in the crystallization (from 0.6 × 0.5 to 4 × 4 mm^2^). This size increase would also imply an increase in its stability since the quality of the crystals is directly related to the loss of water molecules from the crystal surface. The crystals of GdW_30_ grown in the presence of In^3+^ and Y^3+^ form the regular phase of pure GdW_30_ (**P-I**), while the ones grown in the presence of Al^3+^, as well as the family of YW_30_, form two separate phases (**P-II** and **P-III**). The disparity of phases in both systems has pushed us to perform a cocrystallization study of Gd- and Y-POMs in the presence of In^3+^. We observed that YW_30_ can retain its original phase with up to 40% doping of GdW_30_. The effect of In^3+^ in the crystallization of Preyssler-POMs has been further extended to other LnW_30_ systems giving rise to the same trends. This is the first work in which the influence of additive ions on the crystallization of POMs is studied. Due to the possibility not only of obtaining bigger and more stable crystals but also of being able to control their size, these results will have a positive impact on molecular magnetism and quantum computing, as the growth of crystals of sufficient quality is a must for performing these studies.

## Supporting Information Available

Crystallographic data and complementary characterization (PXRD, TGA, and ICP-MS) including detailed images of LnW_30_ crystals and phase index of GdW_30_/YW_30_ (PDF)

## References

[ref1] Pope M. T.; Müller A. Polyoxometalate Chemistry: An Old Field with New Dimensions in Several Disciplines. Angew. Chem., Int. Ed. Engl. 1991, 30, 34–48. 10.1002/anie.199100341.

[ref2] Müller A.; Peters F.; Pope M. T.; Gatteschi D. Polyoxometalates: Very Large Clusters-Nanoscale Magnets. Chem. Rev. 1998, 98, 239–272. 10.1021/cr9603946. 11851505

[ref3] Gumerova N. I.; Rompel A. Synthesis, structures and applications of electron-rich polyoxometalates. Nat. Rev. Chem. 2018, 2, 0112 10.1038/s41570-018-0112.

[ref4] Huang B.; Yang D. H.; Han B. H. Application of polyoxometalate derivatives in rechargeable batteries. J. Mater. Chem. A 2020, 8, 4593–4628. 10.1039/C9TA12679A.

[ref5] Blasco-Ahicart M.; Soriano-López J.; Carbó J. J.; Poblet J. M.; Galan-Mascaros J. R. Polyoxometalate electrocatalysts based on earth-abundant metals for efficient water oxidation in acidic media. Nat. Chem. 2018, 10, 24–30. 10.1038/nchem.2874. 29256497

[ref6] Soriano-López J.; Elliott R.; Kathalikkattil A. C.; Ako A. M.; Mulahmetović M.; Venkatesan M.; Schmitt W. Bioinspired Water Oxidation Using a Mn-Oxo Cluster Stabilized by Non-Innocent Organic Tyrosine Y161 and Plastoquinone Mimics. ACS Sustainable Chem. Eng. 2020, 8 (36), 13648–13659. 10.1021/acssuschemeng.0c03379.

[ref7] Clemente-Juan J. M.; Coronado E.; Gaita-Ariño A. Magnetic polyoxometalates: from molecular magnetism to molecular spintronics and quantum computing. Chem. Soc. Rev. 2012, 41, 7464–7478. 10.1039/c2cs35205b. 22948854

[ref8] Coronado E. Molecular magnetism: from chemical design to spin control in molecules, materials and devices. Nat. Rev. Mater. 2020, 5, 87–104. 10.1038/s41578-019-0146-8.

[ref9] Preyssler C. Bull. Soc. Chim. Fr. 1970, 1, 30–36.

[ref10] Alizadeh M. H.; Harmalker S. P.; Jeannin Y.; Martin-Frère J.; Pope M. T. A Heteropolyanion with Fivefold Molecular Symmetry That Contains a Nonlabile Encapsulated Sodium Ion. The Structure and Chemistry of [NaP_5_W_30_O_110_]^14-^. J. Am. Chem. Soc. 1985, 107, 2662–2669. 10.1021/ja00295a019.

[ref11] Creaser I.; Heckel M. C.; Neitz R. J.; Pope M. T. Rigid Nonlabile Polyoxometalate Cryptates [ZP_5_W_30_O_110_]^(15-n)-^ That Exhibit Unprecedented Selectivity for Certain Lanthanide and Other Multivalent Cations. Inorg. Chem. 1993, 32, 1573–1578. 10.1021/ic00061a010.

[ref12] Cardona-Serra S.; Clemente-Juan J. M.; Coronado E.; Gaita-Ariño A.; Camón A.; Evangelisti M.; Luis F.; Martínez-Pérez M. J.; Sesé J. Lanthanoid single-ion magnets based on polyoxometalates with a 5-fold symmetry: The series [LnP_5_W_30_O_110_]^12-^ (Ln^3+^ = Tb, Dy, Ho, Er, Tm, and Yb). J. Am. Chem. Soc. 2012, 134, 14982–14990. 10.1021/ja305163t. 22894703

[ref13] Martínez-Pérez M. J.; Cardona-Serra S.; Schlegel C.; Moro F.; Alonso P. J.; Prima-García H.; Clemente-Juan J. M.; Evangelisti M.; Gaita-Ariño A.; Sesé J.; van Slageren J.; Coronado E.; Luis F. Gd-Based Single-Ion Magnets with Tunable Magnetic Anisotropy: Molecular Design of Spin Qubits. Phys. Rev. Lett. 2012, 108, 247213 10.1103/PhysRevLett.108.247213. 23004325

[ref14] Gaita-Ariño A.; Luis F.; Hill S.; Coronado E. Molecular spins for quantum computation. Nat. Chem. 2019, 11, 301–309. 10.1038/s41557-019-0232-y. 30903036

[ref15] Jenkins M. D.; Duan Y.; Diosdado B.; García-Ripoll J. J.; Gaita-Ariño A.; Giménez-Saiz C.; Alonso P. J.; Coronado E.; Luis F. Coherent manipulation of three-qubit states in a molecular single-ion magnet. Phys. Rev. B 2017, 95, 064423 10.1103/PhysRevB.95.064423.

[ref16] Mizuno K.; Mura T.; Uchida S. Control of Polymorphisms and Functions in All-Inorganic Ionic Crystals Based on Polyaluminum Hydroxide and Polyoxometalates. Cryst. Growth Des. 2016, 16, 4968–4974. 10.1021/acs.cgd.6b00555.

[ref17] Chen L.; Turo M. J.; Gembicky M.; Reinicke R. A.; Schimpf A. M. Cation-Controlled Assembly of Polyoxotungstate-Based Coordination Networks. Angew. Chem., Int. Ed. 2020, 59, 16609–16615. 10.1002/anie.202005627. 32488927

[ref18] Dolomanov O. V.; Bourhis L. J.; Gildea R. J.; Howard J. A. K.; Puschmann H. OLEX2: A Complete Structure Solution, Refinement and Analysis Program. J. Appl. Crystallogr. 2009, 42, 339–341. 10.1107/S0021889808042726.

[ref19] Sheldrick G. M. SHELXT–Integrated space-group and crystal-structure determination. Acta Crystallogr. 2015, A71, 3–8. 10.1107/S2053273314026370. PMC4283466 25537383

[ref20] Sheldrick G. M. Crystal structure refinement with SHELXL. Acta Crystallogr. C Struct. Chem. 2015, C71, 3–8. 10.1107/S2053229614024218. PMC4294323 25567568

[ref21] Meldrum F. C.; Cölfen H. Controlling Mineral Morphologies and Structures in Biological and Synthetic Systems. Chem. Rev. 2008, 108 (11), 4332–4432. 10.1021/cr8002856. 19006397

[ref22] Song R.; Cölfen H. Additive controlled crystallization. CrystEngComm 2011, 13, 1249–1276. 10.1039/c0ce00419g.

[ref23] Sangwal K. Additives and Crystallization Processes: From Fundamentals to Applications; John Wiley & Sons Ltd: England, 2007.

[ref24] De Yoreo J. J.; Wierzbicki A.; Dove P. M. New insights into mechanisms of biomolecular control on growth of inorganic crystals. CrystEngComm 2007, 9, 1144–1152. 10.1039/b713006f.

[ref25] Elhadj S.; Salter E. A.; Wierzbicki A.; De Yoreo J. J.; Han N.; Dove P. M. Peptide Controls on Calcite Mineralization: Polyaspartate Chain Length Affects Growth Kinetics and Acts as a Stereochemical Switch on Morphology. Cryst. Growth. Des. 2006, 6 (1), 197–201. 10.1021/cg050288+.

