# Supplementary material for: Effect of Nonconstituent Additive Ions on the Controlled Crystallization of Lanthanide-Based Preyssler Polyoxometalates
Source: Cryst Growth Des. 2023 Apr 6;23(5):3544–8. doi: 10.1021/acs.cgd.3c00046 (PMC10161220; doi:10.1021/acs.cgd.3c00046)
Supplement: Supplementary file 1 — cg3c00046_si_001.pdf [file cg3c00046_si_001.pdf]

Supporting Information

Effect of non-constituent additive ions on the controlled crystallization of lanthanide-based Preyssler polyoxometalates

*Iván Gómez-Muñoz,† Chandan Dey,† and Eugenio Coronado**

Instituto de Ciencia Molecular (ICMol), Universidad de Valencia, c/Catedrático José Beltrán 2, Paterna, 46980, Spain.

KEYWORDS: magnetic polyoxometalates, crystal growth, lanthanides, Preyssler structure

†*These authors equally contributed*

1. **Single Crystal X-ray Diffraction**

Table S1. Crystallographic data

Figure S1. Face indexing of the crystals

2. **Effect of the concentration of In^{3+} on the crystallization of YW_{30}**

Figure S2. X-ray powder diffractograms

Table S2. Surface exposure study

Figure S3. Thermogravimetric analysis

3. **Crystallization of other LnW_{30} systems**

Figure S4. Crystal size and morphology

Figure S5. X-ray powder diffraction

Table S3. Elemental analysis

4. **Co-crystallization of YW_{30} and GdW_{30}**

Table S4. Analysis of X-ray data

Figure S6. Phase-II index

Table S5. Study of different concentrations of $InCl_3$

1. Single Crystal X-ray Diffraction

Table S1. Crystallographic data.

Crystal Phase	Phase-I (***P-I***)	Phase-II (***P-II***)	Phase-III (***P-III***)
Empirical formula	$K_{12}GdP_5W_{30}O_{133.5}H_{47}$	$K_{12}YP_5W_{30}O_{138.5}H_{57}$	$K_{11.5}Na_3P_5W_{30}O_{134}H_{48.50}$
Fw	8480.17	8444.46	8381.86
T (K)	120.00(10)	119.9(2)	119.6(8)
λ (Å)	0.71073	0.71073	0.71073
Crystal system	Orthorhombic	Orthorhombic	Monoclinic
Space group	*Pnma*	*Pnna*	$P2_1/m$
a (Å)	28.5872(4)	32.8072(3)	16.8636(2)
b (Å)	21.4797(3)	21.5301(2)	21.0688(2)
c (Å)	20.8888(3)	19.1367(2)	18.0295(2)
α (°)	90	90	90
β (°)	90	90	114.859(2)
γ (°)	90	90	90
V (Å^3)	12826.7(3)	13517.1(2)	5812.276
Z	4	4	2
D_{calc} (g/cm^3)	4.391	4.150	4.789
μ (mm^{-1})	27.858	26.380	30.173
Goodness-of-fit on F^2	1.154	1.071	1.042
R_1 [I > 2σ(I)]	0.0659	0.0681	0.0671
wR_2 [I > 2σ(I)]	0.1635	0.1907	0.1858
R_1 (all data)	0.0734	0.0746	0.0744
wR_2 (all data)	0.1692	0.1993	0.1970

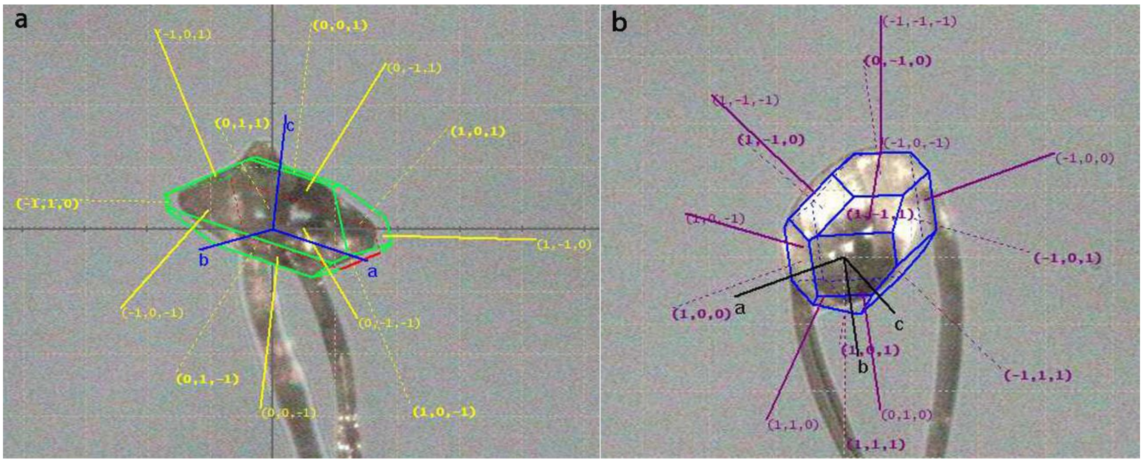

Figure S1. Face indexing of (a) GdW_{30} (b) YW_{30} crystals grown in presence of In^{3+}.

2. Effect of the concentration of In^{3+} on the crystallization of YW_{30}

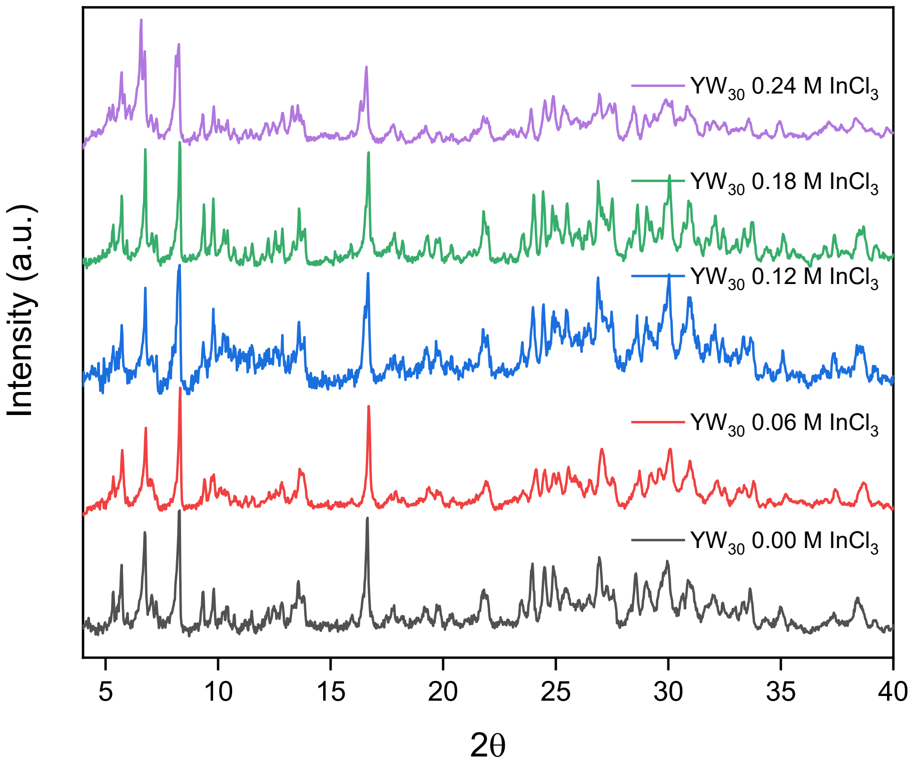

Figure S2. X-ray powder diffraction patterns of YW_{30} grown in presence of different concentrations of $InCl_3$.

Influence of the exposed surface area: The crystallization of YW_{30} was performed in different types of vials and beakers to study the influence of the exposed surface area on the resulting crystal size. In all cases, the concentration of $InCl_3$ was 0.18 M. The results showed that the bigger the exposed surface area is, the faster the evaporation rate and the smaller the crystal size.

Table S2. Influence of the exposed surface area on the average crystal size.

	**Diameter (cm)**	**Height (cm)**	**Volume of the solution (mL)**	**Average crystal size**
**5 mL vial**	1.2	3.6	1.5	5x3 mm^2
**15 mL vial**	1.3	5	1.5	3x4 mm^2
**40 mL vial**	2.2	8	10	1.4x1 mm^2
**25 mL beaker**	3.1	5	10	<1 mm^2

Thermal stability: Thermogravimetric analyses were performed under a nitrogen atmosphere for the samples crystallized in presence of different concentrations of In^{3+}. It has been observed that the rate of decomposition of the compound depends on the average size of the crystalline material. In this way, ground samples exhibit faster decomposition rates than the crude crystals due to having a higher surface area, which eases the solvent loss. The effect of the increased size of the crystals due to the different concentrations of In^{3+} used in the crystallization shows non-consistent results because of the generation of cracks during the process together with the different solvent content of the samples, which is batch dependent.

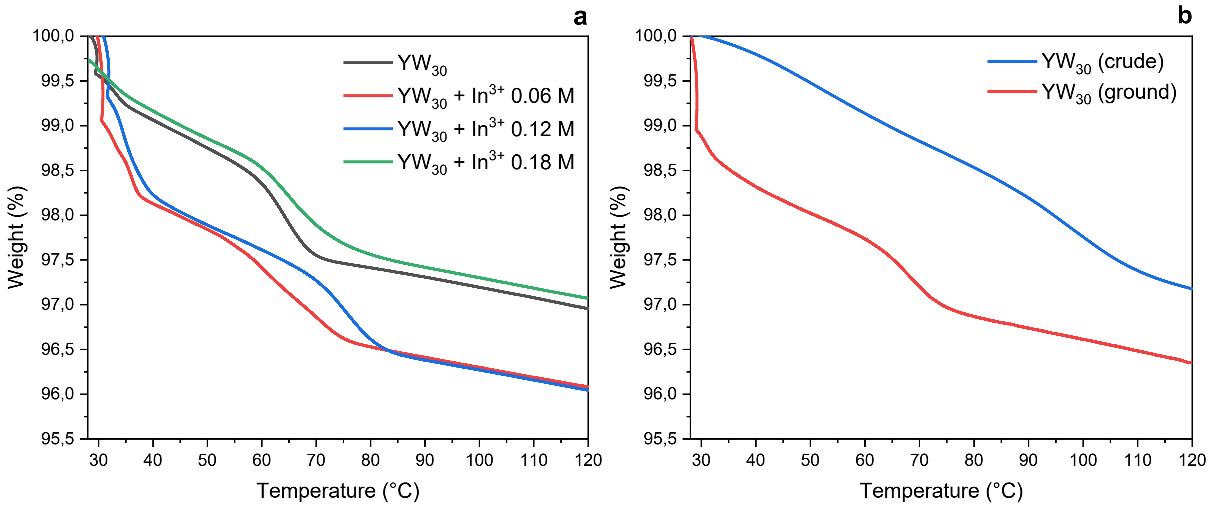

Figure S3. (a) Thermogravimetric analysis of YW_{30} grown in presence of different concentrations of In^{3+}. (b) Comparison of the thermal decomposition of crude (blue) and ground (red) crystals.

3. Crystallization of other LnW_{30} systems

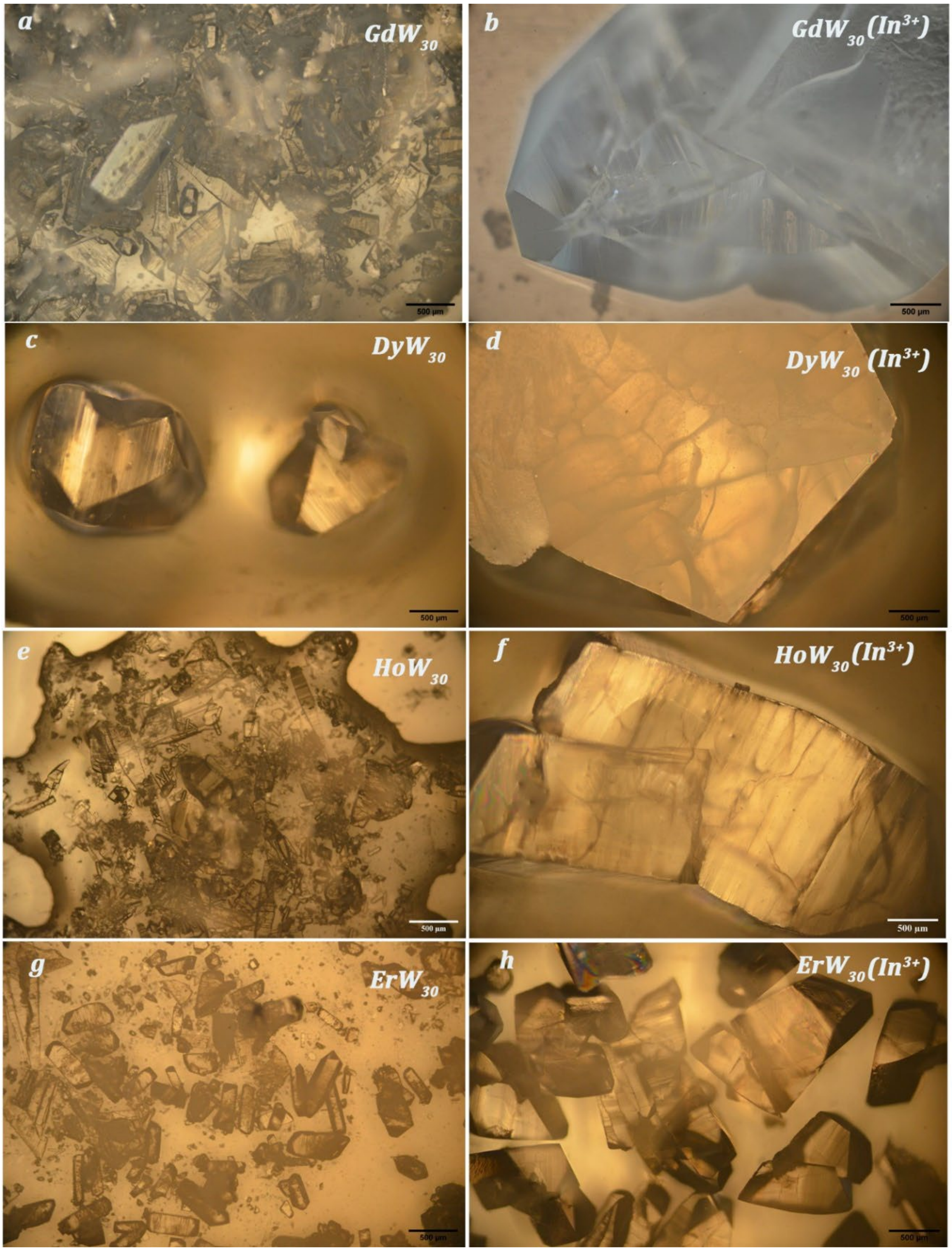

Figure S4. Image of the crystals (a) GdW_{30} without In^{3+} (b) GdW_{30} in presence of 0.12M In^{3+} (c) DyW_{30} without In^{3+} (d) DyW_{30} in presence of 0.12M In^{3+} (e) HoW_{30} without In^{3+} (f) HoW_{30} in presence of 0.12M In^{3+} (g) ErW_{30} without In^{3+} (h) ErW_{30} in presence of 0.12M In^{3+}.

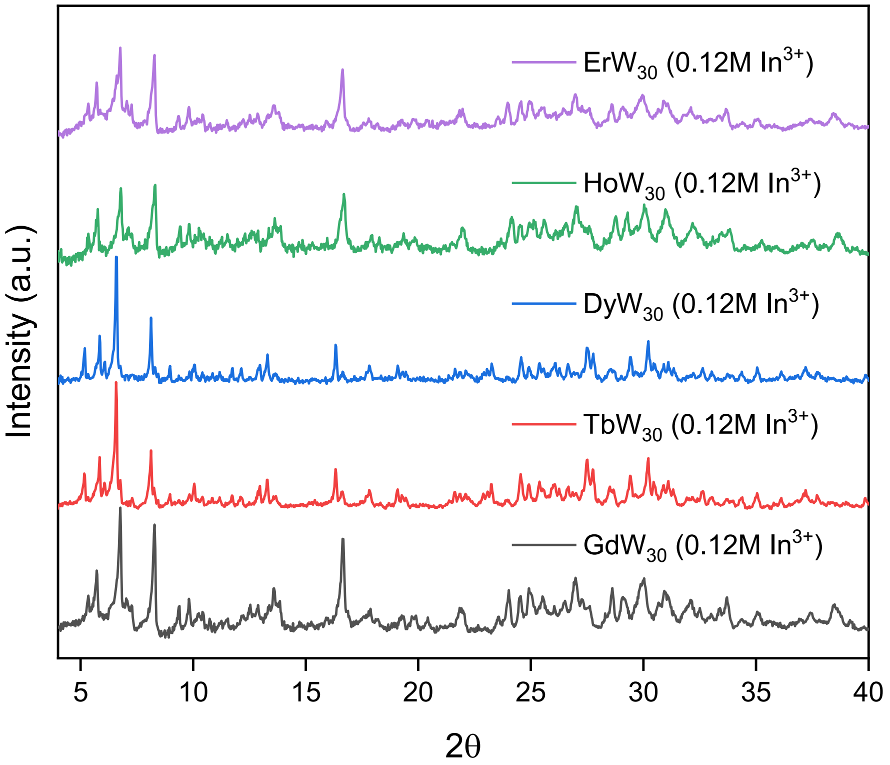

Figure S5. X-ray powder diffractograms of LnW_{30} grown in presence of In^{3+}.

Table S3. Composition of the crystals of LnW_{30} grown in presence of 0.12 M In^{3+}.

**System**	**Composition of crystals grown without In^{3+}**	**Composition of crystals grown in presence of In^{3+}**
GdW_{30}	$K_{12}[GdP_5W_{30}O_{110}]\cdot 40H_2O$	$In_{0.005}Na_{0.3}K_{11.7}[GdP_5W_{30}O_{110}]\cdot 35H_2O$
TbW_{30}	$K_{12}[Tb_{0.92}Na_{0.08}P_5W_{30}O_{110}]\cdot 50H_2O$	$In_{0.08}K_{12}[Tb_{0.9}Na_{0.1}P_5W_{30}O_{110}]\cdot 75H_2O$
DyW_{30}	$K_{12}[Dy_{0.9}Na_{0.1}P_5W_{30}O_{110}]\cdot 40H_2O$	$In_{0.005}K_{12}[Dy_{0.8}Na_{0.2}P_5W_{30}O_{110}]\cdot 70H_2O$
HoW_{30}	$K_{12}[Ho_{0.9}\ Na_{0.1}P_5W_{30}O_{110}]\cdot 25H_2O$	$In_{0.04}K_{12}[Ho_{0.9}Na_{0.1}P_5W_{30}O_{110}]\cdot 30H_2O$
ErW_{30}	$K_{12}[Er_{0.9}\ Na_{0.1}P_5W_{30}O_{110}]\cdot 25H_2O$	$In_{0.03}K_{12}[Er_{0.8}Na_{0.2}P_5W_{30}O_{110}]\cdot 60H_2O$

4. Co-crystallization of YW_{30} and GdW_{30}

Table S4. X-ray data analysis of [GdW_{30}/YW_{30}] grown in presence of 0.2 M $InCl_3$:

**[GdW_{30}/YW_{30}] in solution (%)**	**Phase-II index**	**Composition by ICP-MS**
0 (pure YW_{30})	81.91%	$In_{0.03}H_2K_{10}[Y_{0.7}Na_{0.3}P_5W_{30}O_{110}]\cdot 60H_2O$
12	72.18%	-
16	85.98 %	$In_{0.06}H_{2.5}K_{10}[Y_{0.12}Gd_{0.02}Na_{0.86}P_5W_{30}O_{110}]\cdot 25H_2O$
18	76.30%	$In_{0.05}H_{0.8}K_{13}[Y_{0.11}Gd_{0.03}Na_{0.86}P_5W_{30}O_{110}]\cdot 27H_2O$
20	37.43%	-
22	72.37%	$In_{0.06}H_{2.5}K_{10}[Y_{0.14}Gd_{0.04}Na_{0.82}P_5W_{30}O_{110}]\cdot 30H_2O$
25	58.94%	-
33	69.31%	-
40	41.56%	$In_{0.15}K_{12}[Y_{0.4}Gd_{0.3}Na_{0.3}P_5W_{30}O_{110}]\cdot 35H_2O$

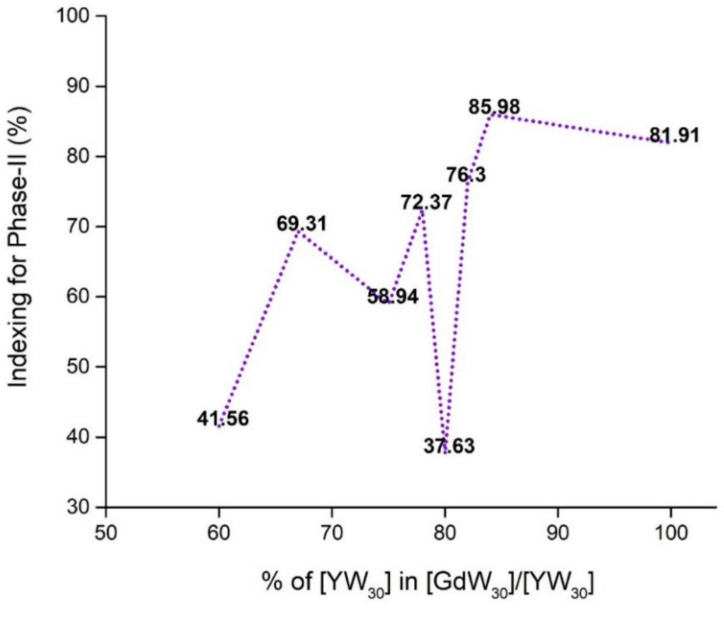

Figure S6. The Phase-II index decreases along with the content of YW_{30} in the system.

Table S5. Crystallization of 40% [GdW_{30}/YW_{30}] in presence of different concentrations of In^{3+}.

**Concentration of In^{3+}**	**Composition**
0.1 M	$In_{0.005}K_{12}[Y_{0.3}Gd_{0.3}Na_{0.4}P_5W_{30}O_{110}]\cdot 30H_2O$
0.2 M	$In_{0.15}K_{12}[Y_{0.4}Gd_{0.3}Na_{0.3}P_5W_{30}O_{110}]\cdot 35H_2O$
0.4 M	$In_{0.06}K_{12}[Y_{0.3}Gd_{0.3}Na_{0.4}P_5W_{30}O_{110}]\cdot 35H_2O$
